# Editorial: Brain-Computer Interfaces and Augmented/Virtual Reality

**DOI:** 10.3389/fnhum.2020.00144

**Published:** 2020-05-12

**Authors:** Felix Putze, Athanasios Vourvopoulos, Anatole Lécuyer, Dean Krusienski, Sergi Bermúdez i Badia, Timothy Mullen, Christian Herff

**Affiliations:** ^1^Cognitive Systems Lab, University of Bremen, Bremen, Germany; ^2^Institute for Systems and Robotics (ISR), Instituto Superior Técnico, Lisbon, Portugal; ^3^Institut National de Recherche en Informatique et en Automatique (INRIA), Rocquencourt, France; ^4^Department of Biomedical Engineering, Virginia Commonwealth University, Richmond, VA, United States; ^5^Madeira Interactive Technologies Institute, University of Madeira, Funchal, Portugal; ^6^Intheon Labs, San Diego, CA, United States; ^7^Department of Neurosurgery, School of Mental Health and Neurosciences, Maastricht University, Maastricht, Netherlands

**Keywords:** BCI, EEG, fNIRS, virtual reality, augmented reality

## 1. Scope

In recent years, Augmented and Virtual Reality (AR/VR) has matured technically, delivering higher levels of immersion and presence to users, while it has also become a widely available tool to create a new range of applications and experiences. AR/VR technology allows to create scenarios which are much more stimulating and expressive than standard desktop applications, covering a wide variety of areas, namely entertainment, education, art, and health, among others.

The fusion of Brain-Computer Interfaces (BCI) with AR/VR can provide additional communication channels, by increasing the bandwidth of the human-AR/VR interaction. This is achieved either explicitly through active BCIs or implicitly using passive BCIs. Active BCIs notably allow users to issue commands to devices or to enter text without physical involvement of any kind, while passive BCIs monitor a user's state (e.g., workload level, attentional state) and can be used to proactively adapt the VR/AR interface. See Lotte et al. (2012) for a general review on mixing VR and BCI technologies.

BCIs, together with AR/VR, offer the possibility for immersive scenarios through induced illusions of an artificially perceived reality that can be utilized not only in basic BCI research but also in many fields of application: In basic research, AR/VR can be used to adjust intensity, complexity, and realism of stimuli smoothly while maintaining full control over their presentation. In therapeutic applications, AR/VR can create motivating training paradigms that make use of gamification approaches or believable illusions, e.g., of virtual limbs interacting with the environment. In Human-Computer Interaction, AR/VR can be used for rapid prototyping of new interface paradigms in realistic settings. To live up to these expectations, methodological advances are required for BCI interaction and stimulus design, synchronization, or dealing with VR/AR specific artifacts (Tremmel et al., 2019) and distractions.

The papers in this Research Topic show both the upsides and the potential of BCI research for or with AR/VR technology, as well as the challenges that lie on the way to such an integration. Moreover, both passive and active BCIs are presented from current studies. In terms of passive BCIs, attentional state in AR is investigated by Vortmann et al. and workload assessment in VR by Tremmel et al.. In terms of active BCIs, virtual avatar arms are controlled directly in motor-imagery BCI training for neurorehabilitation by Vourvopoulos, Pardo et al., Vourvopoulos, Jorge et al. and also in a more gamified training by Škola et al.

Overall, we see that BCI can be used in VR/AR-based training for workload and attention assessment, but also in rehabilitation contexts, where the VR components creates a much more immersive experience compared to traditional training paradigms.

## 2. Research Highlights

In the Research Topic, we find works both on passive and active BCI technology. Regarding passive BCI, research concentrates on two aspects which are often tackled for adaptive technology: attention and workload. From their motivation, these papers aim for an improvement of the AR/VR interface itself through user state adaptation.

Vortmann et al. perform a study on the classification of internal from external attention in an AR setting. The authors develop a novel AR task requiring continuous spatial alignment, mimicking typical AR interactions. They show that using frequency features, the classifier achieves an average classification accuracy of 85% using windows of 13 s length. Recently, they demonstrated an real time implementation of the attention model (Vortmann et al., 2019), enabling online adaptation of AR-based user interfaces, such as a smart home control in AR (Putze et al., 2019b) using Steady-State Visually Evoked Potentials (SSVEP) and eye tracking to select from virtual menus displayed in the environment. Other related work is by Si-Mohammed et al. (2018), a pioneer study on the feasibility and design of 3D User Interfaces exploiting AR for SSVEP-based BCI.

Tremmel et al. perform a study to measure mental workload in VR. For this purpose, they adapted the well-studied *n*-back task for interactive VR and made it publicly available. They recorded neural activity, recorded through Electroencephalography (EEG), from 15 participants performing 0-, 1-, and 2-back trials and could show that workload levels could be discriminated from the scalp recordings despite the large level of physical movement occuring during VR usage. Additionally, they showed the feasibility of using functional Near Infrared Spectroscopy (fNIRS) as an alternative modality (Putze et al., 2019a), as both fNIRS (Herff et al., 2014) and the combination of fNIRS and EEG (Herff et al., 2015) have been shown to be successful in workload classification. This opens the door for future multimodal systems on workload classification in VR.

Other papers concentrate on the integration of active BCI technology in VR settings. In contrast to the passive BCI contributions, this research is less about the potential improvement of the VR interface, but about leveraging this immersive technology to improve traditional BCI paradigms, for example in rehabilitation.

Vourvopoulos, Pardo et al. performed a study by combining the principles of VR and BCI in a rehabilitation training platform called REINVENT (Vourvopoulos et al., 2019), assessing its effects on four chronic stroke patients across different levels of motor impairment. The acquired post-stroke EEG signals that indicate an attempt to move, drive a virtual avatar arm (from the affected side), allowing patient-driven action observation BCI in VR. They show that EEG-based BCI-VR may benefit patients with more severe motor impairments, by increasing their communication bandwidth to VR, in-contrast to patients with more mild impairments that they can still harness existing sensorimotor pathways with EMG-based feedback in the same VR training (Marin-Pardo et al., 2019).

By extending BCI-VR training from uni-manual (of the affected arm) into bi-manual control, Vourvopoulos, Jorge et al. performed a study by using NeuRow, a self-paced BCI paradigm in VR (Vourvopoulos et al., 2016) together with brain-imaging data (fMRI) in a chronic stroke patient. They found important improvements in upper extremity clinical scales (Fugl-Meyer) and identified increases in brain activation measured by fMRI that suggest neuroplastic changes in brain motor networks. These results suggest that BCI with ecologically-valid VR could be useful in chronic stroke patients with reduced upper limb motor function while together with brain-imaging data we move toward identifying the specific benefits of brain-controlled VR training environments for neurorehabilitation.

Finally, Škola et al. presented a study with a gamified BCI-VR training including virtual avatar designed with the aim to maintain high levels of attention and motivation. This was achieved using a progressively increasing training, event-driven and not self-paced by providing participants with score points about their progress. Performance levels in terms of classifier performance show higher than chance level (65%) and the strength of Event-Related Desynchronizations (ERDs) during session was positively correlated to the subjective magnitude of the sense of ownership while the perceived ownership of the avatar body was not correlated to the BCI performance nor to the sense of agency.

## 3. Summary

The presented articles present the large potential of combining AR/VR technology with BCIs to further increase the immersiveness of AR/VR and improve the usability of BCIs for rehabilitation and control. All presented papers use elaborate, task-specific experiment setups with custom AR/VR environments and custom solutions to set up sensors, input devices, and displays [see (Putze, 2019) for an overview of emerging methods and tools and (Si-Mohammed et al., 2017) for a general overview of potential applications and main scientific challenges related to the combination of AR and BCI]. Future research can build on this pioneering works and the resulting best practices to derive more standardized, common experiment protocols for a large variety of research questions. This would lower the entry barrier and make the promising technology more accessible.

## Author Contributions

FP created the structure and initial draft. CH, AV, and FP contributed the paper summaries. All authors contributed to the overview of the field.

## Conflict of Interest

TM is CEO & Research Director of Intheon Labs. The remaining authors declare that the research was conducted in the absence of any commercial or financial relationships that could be construed as a potential conflict of interest.

## References

[B1] Herff C. Fortmann O. Tse C.-Y. Cheng X. Putze F. Heger D. (2015). Hybrid fNIRS-EEG based discrimination of 5 levels of memory load, in 2015 7th International IEEE/EMBS Conference on Neural Engineering (NER) (IEEE), 5–8.

[B2] Herff C. Heger D. Fortmann O. Hennrich J. Putze F. Schultz T. (2014). Mental workload during n-back task–quantified in the prefrontal cortex using fNIRS. Front. Hum. Neurosci. 7:935. 10.3389/fnhum.2013.00935 24474913 PMC3893598

[B3] Lotte F. Faller J. Guger C. Renard Y. Pfurtscheller G. Lécuyer A. (2012). Combining BCI with virtual reality: towards new applications and improved BCI, in Towards Practical Brain-Computer Interfaces, eds Allison B. Z. Dunne S. Leeb R. Millán J. D. R. Nijholt A. (Berlin; Heidelberg: Springer), 197–220.

[B4] Marin-Pardo O. Vourvopoulos A. Neureither M. Saldana D. Jahng E. Liew S.-L. (2019). Electromyography as a suitable input for virtual reality-based biofeedback in stroke rehabilitation, in HCI International 2019, ed Stephanidis C. (Cham: Springer International Publishing), 274–281.

[B5] Putze F. (2019). Methods and tools for using BCI with augmented and virtual reality, in Brain Art ed. Nijholt A. (Berlin; Heidelberg: Springer), 433–446.

[B6] Putze F. Herff C. Tremmel C. Schultz T. Krusienski D. J. (2019a). Decoding mental workload in virtual environments: a fNIRS study using an immersive n-back task, in 2019 41st Annual International Conference of the IEEE Engineering in Medicine and Biology Society (EMBC) (Berlin: IEEE), 3103–3106. 10.1109/EMBC.2019.8856386 31946544

[B7] Putze F. Weiβ D. Vortmann L.-M. Schultz T. (2019b). Augmented reality interface for smart home control using SSVEP-BCI and eye gaze, in 2019 IEEE International Conference on Systems, Man and Cybernetics (SMC) (Bari: IEEE), 2812–2817.

[B8] Si-Mohammed H. Argelaguet F. Casiez G. Roussel N. Lécuyer A. (2017). Brain-computer interfaces and augmented reality: a state of the art, in Graz Brain-Computer Interface Conference (Graz).

[B9] Si-Mohammed H. Petit J. Jeunet C. Argelaguet F. Spindler F. Evain A.. (2018). Towards BCI-based interfaces for augmented reality: feasibility, design and evaluation. IEEE Trans. Vis. Comput. Graph. 26, 1608–1621. 10.1109/TVCG.2018.2873737 30295623

[B10] Tremmel C. Herff C. Krusienski D. J. (2019). EEG movement artifact suppression in interactive virtual reality, in 2019 41st Annual International Conference of the IEEE Engineering in Medicine and Biology Society (EMBC) Berlin, 4576–4579. 10.1109/EMBC.2019.8856961 31946883

[B11] Vortmann L.-M. Schult M. Benedek M. Walcher S. Putze F. (2019). Real-time multimodal classification of internal and external attention, in Adjunct of the 2019 International Conference on Multimodal Interaction (Suzhou), 1–7.

[B12] Vourvopoulos A. Ferreira A. i Badia S. B. (2016). Neurow: an immersive VR environment for motor-imagery training with the use of brain-computer interfaces and vibrotactile feedback, in Proceedings of the 3rd International Conference on Physiological Computing Systems - Volume 1: PhyCS (Lisbon: INSTICC; SciTePress), 43–53.

[B13] Vourvopoulos A. Marin-Pardo O. Neureither M. Saldana D. Jahng E. Liew S.-L. (2019). Multimodal head-mounted virtual-reality brain-computer interface for stroke rehabilitation, in Virtual, Augmented and Mixed Reality. Multimodal Interaction, eds Chen J. Y. Fragomeni G. (Cham: Springer International Publishing), 165–179.

